# Contrasts and similarities in the transcriptomic response to antimicrobial coinage metals in *Escherichia coli*

**DOI:** 10.1128/spectrum.02541-25

**Published:** 2026-06-15

**Authors:** Daniel A. Salazar-Alemán, Ashley McGibbon, Raymond J. Turner

**Affiliations:** 1 Department of Biological Sciences, University of Calgary 2129, Calgary, Alberta, Canada; University of Guelph College of Biological Science, Guelph, Ontario, Canada

**Keywords:** transcriptomics, silver, copper, gold, antimicrobial tolerance, metal resistance, metal-based antimicrobials, iron metabolism, thiol metabolism, cell envelope, metal efflux, zinc import

## Abstract

With the rise of resistance to last-resort antibiotics, metal-based antimicrobials have re-emerged as an alternative to prevent and manage infections. The group 11 metals (copper, silver, gold), historically known for their usage in coins, have demonstrated promising bactericidal activity. Despite their efficiency, we do not have a complete understanding of how bacteria are eradicated by metal ions and how they respond to metal-induced stress. Additionally, most studies in the field focus on the physiological response to acute toxicity, often overlooking longer exposure models. We used RNA-seq profiling to understand the *Escherichia coli* physiological response to sublethal inhibitory antimicrobial coinage metal stress after 10 hours of incubation. Gene expression patterns of the adaptive and intrinsic response elicited by each metal were identified, including increased essential metal uptake (Ag, Cu, Au), cysteine biosynthesis (Cu, Au), change of the metal ion oxidation state (Cu, Au), efflux of metal stressor (Cu), protein translation, and ribosome biogenesis (Au), and cell envelope stress response (Ag). We highlight the remarkable differences and similarities in the transcriptomic response profile of *E. coli* to these antimicrobial metal elements.

**IMPORTANCE:**

Dogma existed in the past, stating that all antimicrobial metals kill bacteria the same way. Thus, the assumption was that bacteria respond the same way to metal toxicity. Nowadays, we understand better why some metal elements are more toxic than others, but questions remain in relation to how bacteria adapt to survive and thrive when challenged by different metal-based antimicrobials. Our study advances the field by characterizing the type of bacterial response needed to acclimate and grow in the presence of silver, copper, and gold—metallic elements known for their antimicrobial activity. Taking advantage of well-characterized *Escherichia coli*, we propose a model that summarizes our findings after comparing the shared and unique responses to each of these metals. This information enhances our understanding of bacterial tolerance to metal-based antimicrobials, which can lead to improved drug development strategies as society continues to search for alternatives against antibiotic-resistant pathogens.

## INTRODUCTION

Metal-based antimicrobials (MBAs) have been used for millennia. Ancient civilizations, such as the Persians and Phoenicians, used silver and copper in vessels to prevent water fouling (1). British settlers in America preserved water by suspending precious metal coins in wooden casks (2). The “father of bacteriology,” Robert Koch, explored the effectiveness of silver and cyanide-gold compounds against *Mycobacterium tuberculosis* through the last two decades of the 19th century (3). Antimicrobial approaches that harnessed the biocidal properties of metals enjoyed the spotlight until Sir Alexander Fleming’s discovery of penicillin in 1928 (4), which subsequently caused antibiotics to become widely used. Nowadays, with the increasing incidence of bacterial strains resistant to most classes of antibiotics (5), MBAs have re-emerged as an alternative to prevent and manage infections (6).

Silver, copper, and gold are group 11 transition metal elements. These are considered precious and thus have been historically used to mint coins; hence the term *coinage metals*. Their effectiveness (7–12) against different WHO critical priority pathogens (5) has led them to return in popularity as antimicrobials. Silver-based antimicrobials are commercially available and see applications in health, including coatings for medical indwelling devices (13), as topical wound dressings (14), and impregnated on textile fabrics (15). Copper is also used in medical settings as part of coatings for high-touch surfaces (10), medical devices (16), and in textiles (17), in addition to a wide variety of usages in agriculture, animal husbandry, and aquaculture due to its antifouling, algaecide, antifungal, and crop-enhancing properties (18). Gold complexes and nanoparticles are under research for their anticancer (19, 20) and antiarthritic (21) properties beyond their bactericidal activity (11, 22, 23), making them good candidates for drug repurposing (24).

We now have a reasonably good understanding of metal toxicity mechanisms and resistance determinants that have allowed further exploration into developing novel MBAs (25–29). Under a One Health perspective, scientists and society must ponder the downstream effects of increased MBA use. The rise of resistance toward metals in the environment is a concern as more MBA applications hit the market, a consequence of the acclimation and passive selection of tolerant organisms that find a way to thrive at exposures below lethal concentrations. Instances of co-selection of resistant determinants to both metal stressors and organic antibiotics are now observed (30). Thus, there is a need to look beyond the standard acute toxicity models (which focus on effective and rapid killing) and investigate changes in bacterial physiology when exposed to sublethal concentrations of antimicrobials, and thus how a culture adapts to such a stress.

In this context, here we compare *Escherichia coli* bacterial physiology after 10 hours of growth in the presence of sublethal concentrations of silver nitrate (AgNO_3_—hereby referred to by the elemental symbol Ag), copper sulfate (CuSO_4_–Cu), and tetrachloroauric acid (HAuCl_4_–Au). This is achieved by leveraging three different RNA-seq data sets and interrogating the *E. coli* transcriptional regulatory network based on gene expression data. As a result, we present the first transcriptomic comparative analysis of the adaptive and intrinsic response profiles upon acclimation to antimicrobial coinage metal-induced stress. Common and unique gene expression patterns of the response elicited by each metal salt were identified, including increased essential metal uptake (Ag, Cu, Au), cysteine biosynthesis (Cu, Au), change of the metal ion oxidation state (Cu, Au), efflux of metal stressor (Cu), translation and ribosome biogenesis (Au), and cell envelope stress response (Ag).

## RESULTS

### Sublethal inhibitory concentration determination

Susceptibility assays were performed on *E. coli* K12 BW25113. Concentrations of 7, 39, and 10 µM of Ag, Cu, and Au, respectively, were deemed as the sublethal inhibitory concentrations to use for the RNA-seq experiments (Fig. S1 through S4). These concentrations exerted a mild inhibitory effect on each bacterial culture as they acclimated to grow in the presence of their respective metal challenges.

### RNA-seq data processing

After processing the sequencing files, differential expression for each metal treatment was obtained by contrasting against their respective non-metal controls. From a total of 4,378 coding sequences in the *E. coli* K12 BW25113 genome assembly, Ag yielded 131 up-regulated differentially expressed genes (UP DEGs) and 558 down-regulated differentially expressed genes (DOWN DEGs); Cu had 80 UP DEGs and 76 DOWN DEGs, while Au had 1,028 and 1,025, respectively (Tables S1 through S3, Fig. S5 through S7). Principal component analysis was performed on the top 5% most variable genes per data set after r-log transformation of their expression data, showing clusters of samples according to the treatment used (Fig. S1).

**Fig 1:**
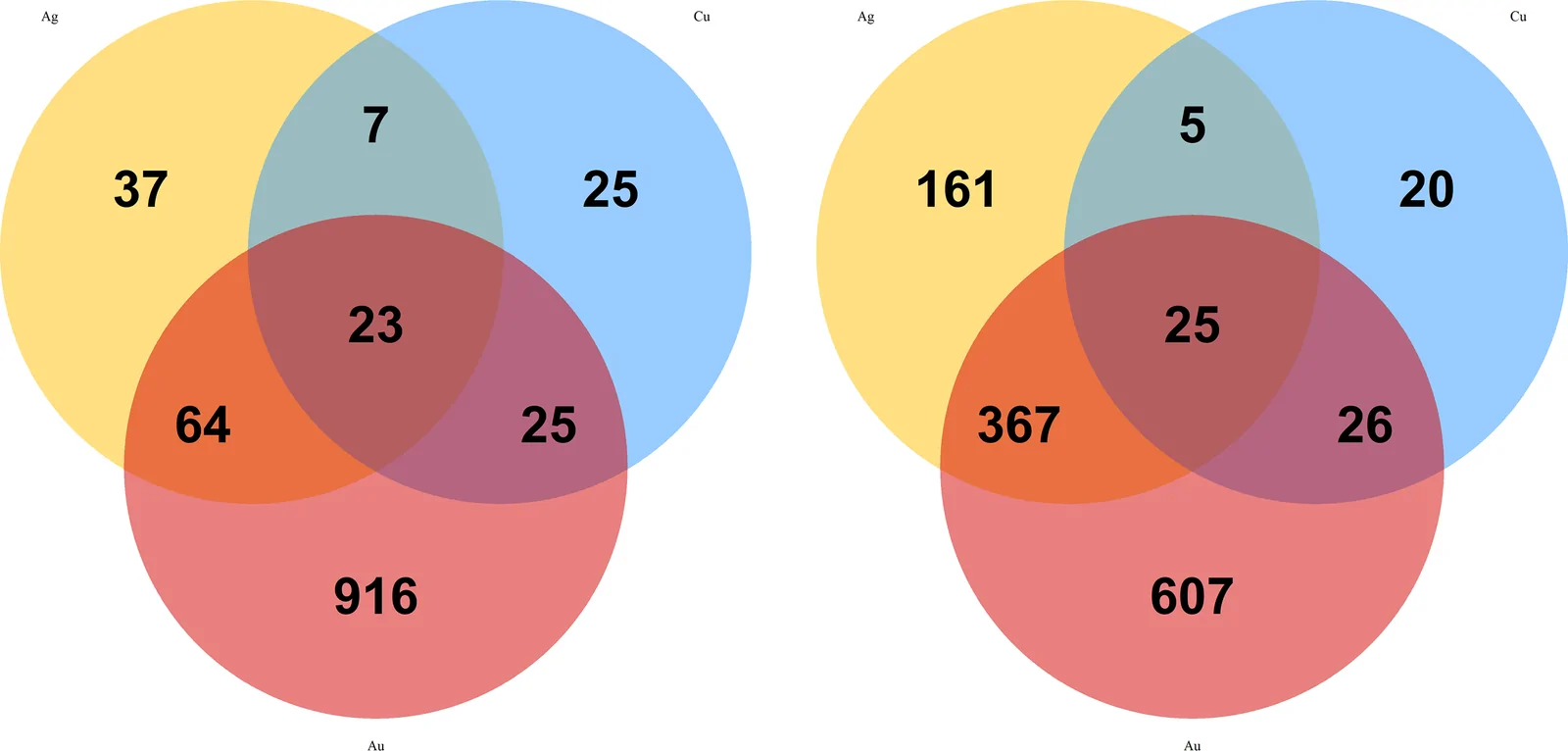
Venn diagrams showing overlaps in up-regulated (left) and down-regulated (right) differentially expressed genes of *E. coli* K12 BW25113 after growing in sublethal concentrations of silver nitrate-, copper sulfate-, or tetrachloroauric acid-spiked M9-glucose minimal media for 10 hours. The yellow circle belongs to the genes from the silver nitrate treatment, blue is for copper sulfate, and red is for tetrachloroauric acid.

We generated Venn diagrams to visualize the overlaps in DEGs from our three metal salt data sets (Fig. 1). Unique DEGs were found in each of our treatments, while also indicating small groups of shared genes between the different metal stressors. Across the three metal salt treatments, 23 genes were shared as UP DEGs (Table 1), while 25 were shared as DOWN DEGs.

**TABLE 1:** List of the 23 significantly up-regulated genes that overlapped across the silver nitrate, copper sulfate, and tetrachloroauric acid RNA-seq data sets

Gene	Product	Uniprot ID
*ybfA*	YbfA family protein	P0AAU2
*ybiE*	Protein YbiE	P0DSE7
*ndh*	NADH-quinone dehydrogenase	P00393
*bhsA*	Multiple stress resistance protein BhsA	P0AB40
*azoR*	FMN-dependent NADH-azoreductase	P41407
*ydeA*	L-arabinose MFS transporter	P31122
*ydhR*	Monooxygenase	P0ACX3
*tcyP*	L-cystine/L-cysteine shuttle transport system	P77529
*guaB*	IMP dehydrogenase	P0ADG7
*grcA*	Autonomous glycyl radical cofactor GrcA	P68066
*serA*	Phosphoglycerate dehydrogenase	P0A9T0
*yrbN*	Protein YrbN	C1P618
*feoA*	Ferrous iron transporter A	P0AEL3
*feoB*	Fe(2+) transporter permease subunit FeoB	P33650
*feoC*	[Fe–S]-dependent transcriptional repressor FeoC	P64638
*glnA*	Glutamate–ammonia ligase	P0A9C5
*zraP*	Zinc responsive periplasmic chaperone ZraP	P0AAA9
*psiE*	Phosphate-starvation-inducible protein PsiE	P0A7C8
*xylE*	D-xylose transporter XylE	P0AGF4
*yjcB*	YjcB family protein	P32700
*proP*	Glycine betaine/L-proline transporter ProP	P0C0L7
*fecB*	Fe(3+) dicitrate ABC transporter substrate-binding protein FecB	P15028
*fecA*	Fe(3+) dicitrate transport protein FecA	P13036

### Obtaining biological significance by interrogating regulons of interest and co-expression patterns

To further investigate the biological context of the gene expression data, we uploaded our raw FASTQ sequencing files to the Integrated System for Motif Activity Response Analysis (ISMARA) (31) web server. With this bioinformatic tool, we were able to identify the regulons that had the highest and lowest gene expression activity in our metal salt treatments (Tables S1 through S3), as it detects motifs in the promoters of the *E. coli* K12 genome and infers the activity of gene regulators based on the expression of their target genes. One of the regulons that scored among the top 10 in activity across the three treatments is Fur, whose target genes are known to be involved in maintaining iron homeostasis. Another highlight was the presence of some known copper homeostasis mechanisms, governed by the CusR and CueR regulons, with the latter having high activities in the Cu and Au data sets but not in Ag. Full results (Z-score and rank per treatment) are included in Table S7.

**Fig 2:**
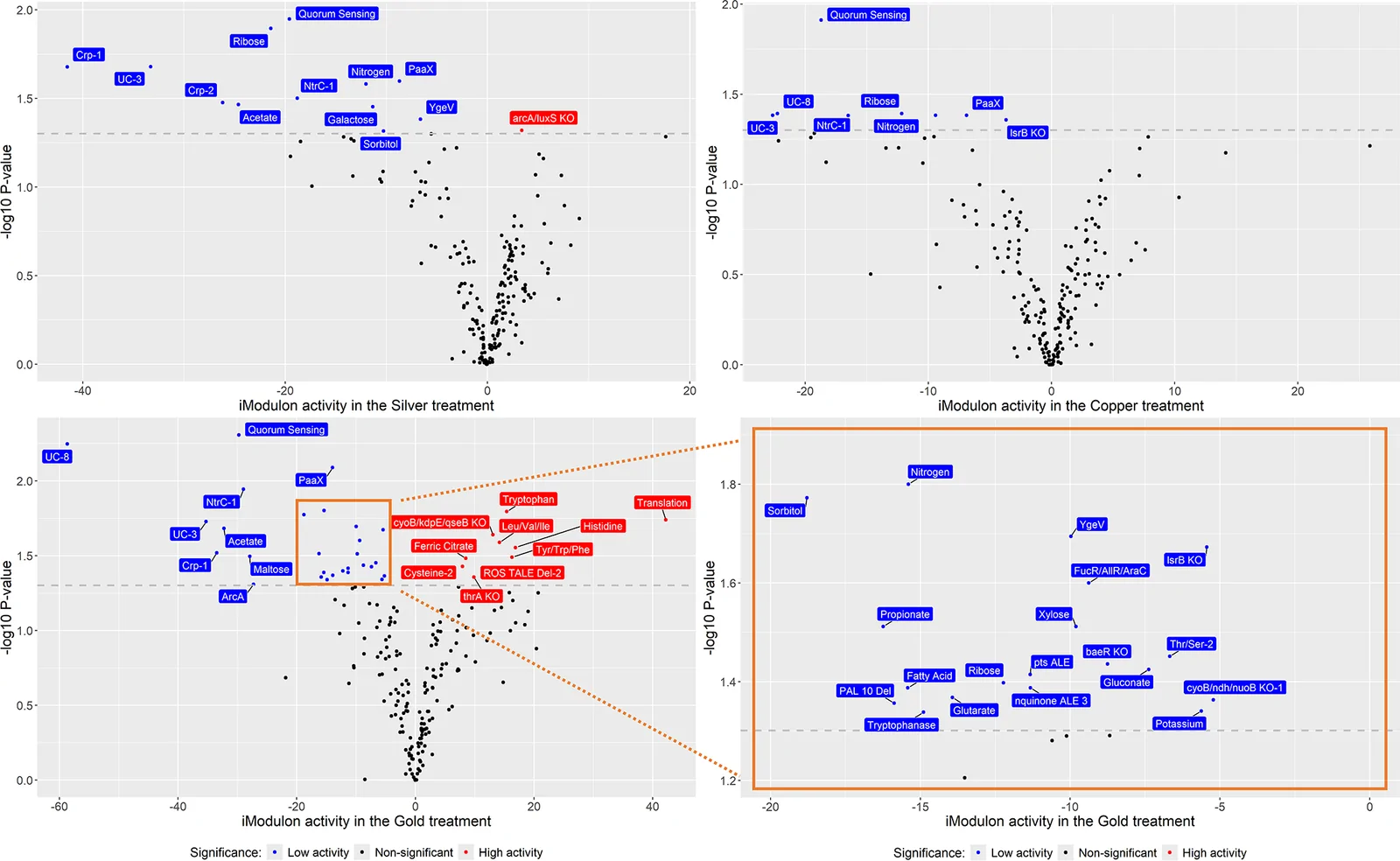
Distribution of iModulon activities in the silver nitrate (top left), copper sulfate (top right), and tetrachloroauric acid (bottom left and right) data sets relative to their non-metal controls. Positive activities in the x-axis indicate iModulons that are more active in the metal condition compared to the control, and vice versa. Statistical significance was calculated using the cumulative log-normal distribution of each iModulon across all conditions. The highlighted data points correspond to iModulons with a *P* value score lower than 0.05.

To identify co-expression patterns, we queried an *E. coli* transcriptional regulatory network based on independently modulated signals, known as iModulons (32). For this purpose, we utilized the *E. coli* PRECISE-1K data set (33). This is composed of 201 groups of independently modulated groups of genes identified by an unsupervised machine-learning algorithm that have shown clear co-expression patterns across 533 different experimental conditions. Each iModulon is subsequently linked to the effect of a single, multiple, or no known regulators. After determining the expression activities for each of the 201 iModulons in our experimental conditions (Fig. 2), we discovered that the Au treatment yielded the highest amount of significant iModulons, with 38; meanwhile, Ag and Cu reported 13 and 8 significant iModulons, respectively. There were no shared significantly up-regulated iModulons across the three metal treatments, which is an indicator of how each metal stressor required a different type of response for the cultures to reach acclimation.

## DISCUSSION

**Fig 3:**
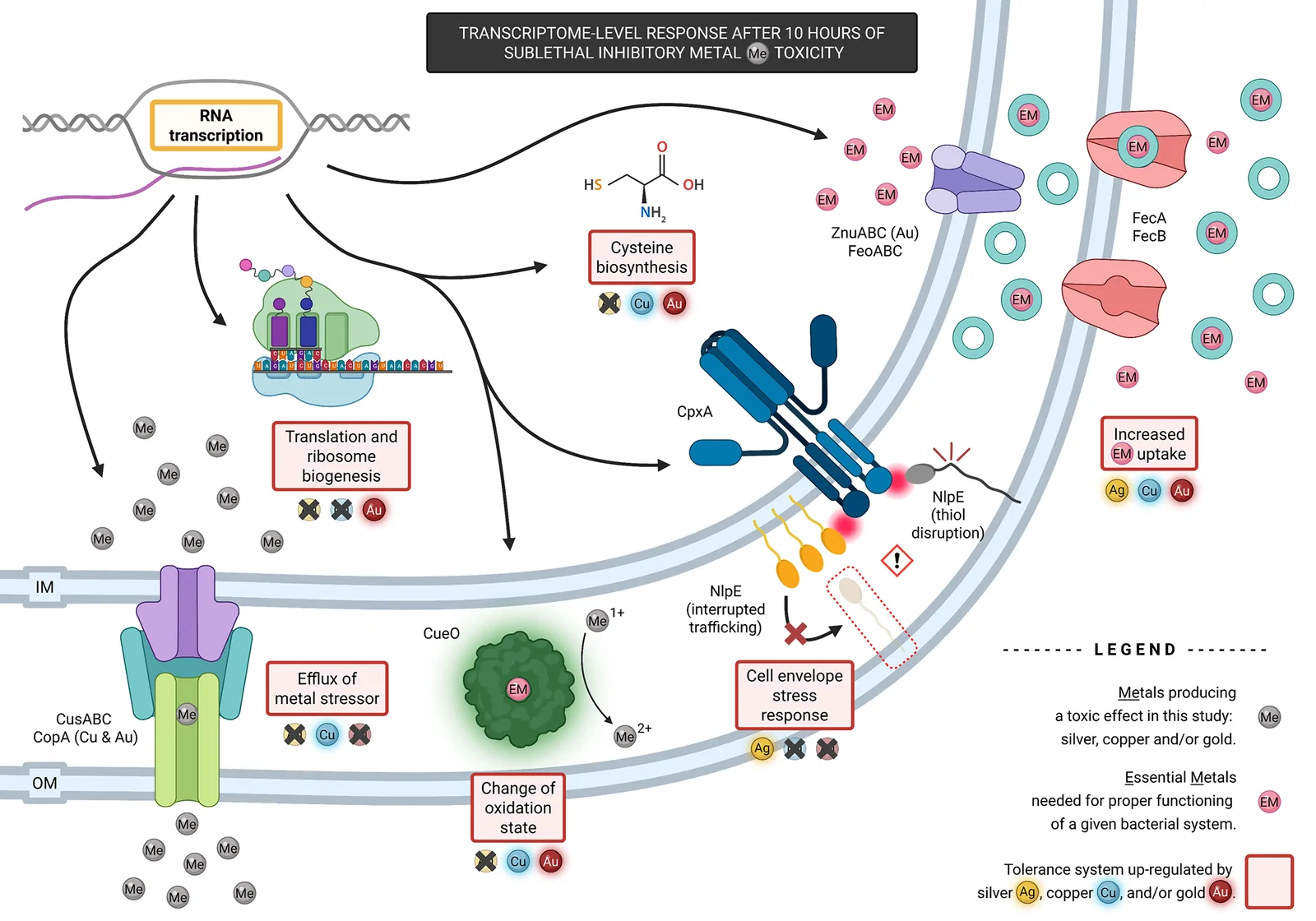
Generalized model of the *E. coli* K12 BW25113 transcriptome-level response to sublethal inhibitory stress induced by antimicrobial coinage metal salts after 10 hours of growth. An increase in the expression of essential metal (EM) uptake systems was observed: iron metabolism and uptake were affected by each metal, while the ZnuABC zinc uptake system was up-regulated by gold (Au). Silver (Ag) triggered the cell envelope stress response, which acts upon outer membrane protein trafficking disruption and misfolding (through the NlpE sentry protein [36]). Copper (Cu) activated the expression of the Cus system, which effluxes excess copper ions. The other copper homeostasis system in *E. coli*, Cue, is sensitive to monovalent metal cations (37) and was up-regulated by Au and Cu: CueO specializes in changing the oxidation state of the more toxic Cu(I) to the less toxic Cu(II), while CopA exports Cu(I) to the periplasm. Cysteine biosynthesis and sulfur metabolism were induced by Cu and Au to replenish the antioxidant pool of the cell and maintain redox balance in the cytoplasm and periplasm. Au also caused increased gene expression activity in ribosome biogenesis and protein translation-related genes. Created with BioRender.com.

Bacterial susceptibility studies have traditionally focused on acute toxicity models, where the focus is to measure lethality and response over short periods of time (0 to 4 hours) after exposing a growing culture to a bactericidal concentration of an antimicrobial. A reflection of this approach is that one typically sees a general SOS stress response highlighting their findings (34). This has the downside effect of ignoring longer exposure models where bacterial physiology is modified as the organism acclimates to the antimicrobial stress. Nowadays, as the scientific community grows aware of the One Health perspective (35), these physiological adaptations that lead to antimicrobial tolerance in prolonged exposure conditions have gained interest. Here, we analyze the adaptive gene expression of *E. coli* growing under sublethal inhibitory antimicrobial coinage metal stress. By leveraging regulon expression analysis and machine learning-based clusters of co-expressed genes, we compared shared and unique trends in the bacterial physiology acclimated to sustained Ag, Cu, and Au-induced stress. Our findings are summarized in Fig. 3 and discussed below.

These elements belong to group 11 of the periodic table and thus share certain properties, but key differences lead to large changes in their interactions with biomolecules. The metal element toxicity level correlates with its physicochemical parameters. A study on *Pseudomonas fluorescens* found that six parameters were correlated to experimental toxicity levels: the metal–sulfide solubility product (pK_sp_), standard redox potential (∆E^0^), electronegativity (X_m_), electron density (Log AR/AW), Pearson's softness index (σ_p_), and covalent index (X_m_^2^r) (38). Table 3 lists these and other physicochemical properties of the three elements under study, which relate to how much toxic stress can be generated based on how the metal ions can interact with biomolecules.

As one goes down group 11 in the periodic table (copper, silver, gold), ionization energy and atomic radius tend to increase, inverse to redox chemistry, which tends to decline to the point that gold is often considered chemically inert (39). Typically, the enthalpies of atomization of the elements increase the lower one goes in each group, but differences tend to be small in the enthalpies between the 3d, 4d, and 5d orbitals of these group 11 elements, reflecting similar strengths of bonding. However, the *ns*^1^ outer electron of these coinage metals can be ionized at higher energies than the alkali metals. Copper and silver have similar first ionization energies at ~740 kJ/mol, but gold is higher at 890 kJ/mol. The pK_sp_ relates to the strength of binding to R-SH groups. These parameters would reflect the on-off bonding rates with the various soft biochemical bases. Other parameters relate to how tight and flexible their ligand coordination geometry is, allowing them to compete out essential metals, leading to a mis-metalation style of toxicity (40). The differences expressed in the values in Table 3 would give an expected order of toxicity of the coinage metal elements as copper > silver > gold. Yet, our results show that silver is the most toxic in *E. coli* (Fig. S2–S4) because cells have evolved important dedicated homeostasis systems to protect cellular targets from free copper ions (discussed below), which do not exist for the other two. Regardless, we would expect the Cu^+^, Ag^−^, and Au^−^ ion forms to be more reactive than their Cu^2+^, Ag^+^, and Au^3+^ or Au^+^ forms of copper, silver, and gold, respectively. Thus, their toxicity may depend more on the redox potential and pH of the cell (in relation to the Pourbaix diagram for each element), which would dictate the relative concentration of the lower oxidation state ions.

### Coinage metal salts affect the expression of iron homeostasis genes

**Fig 4:**
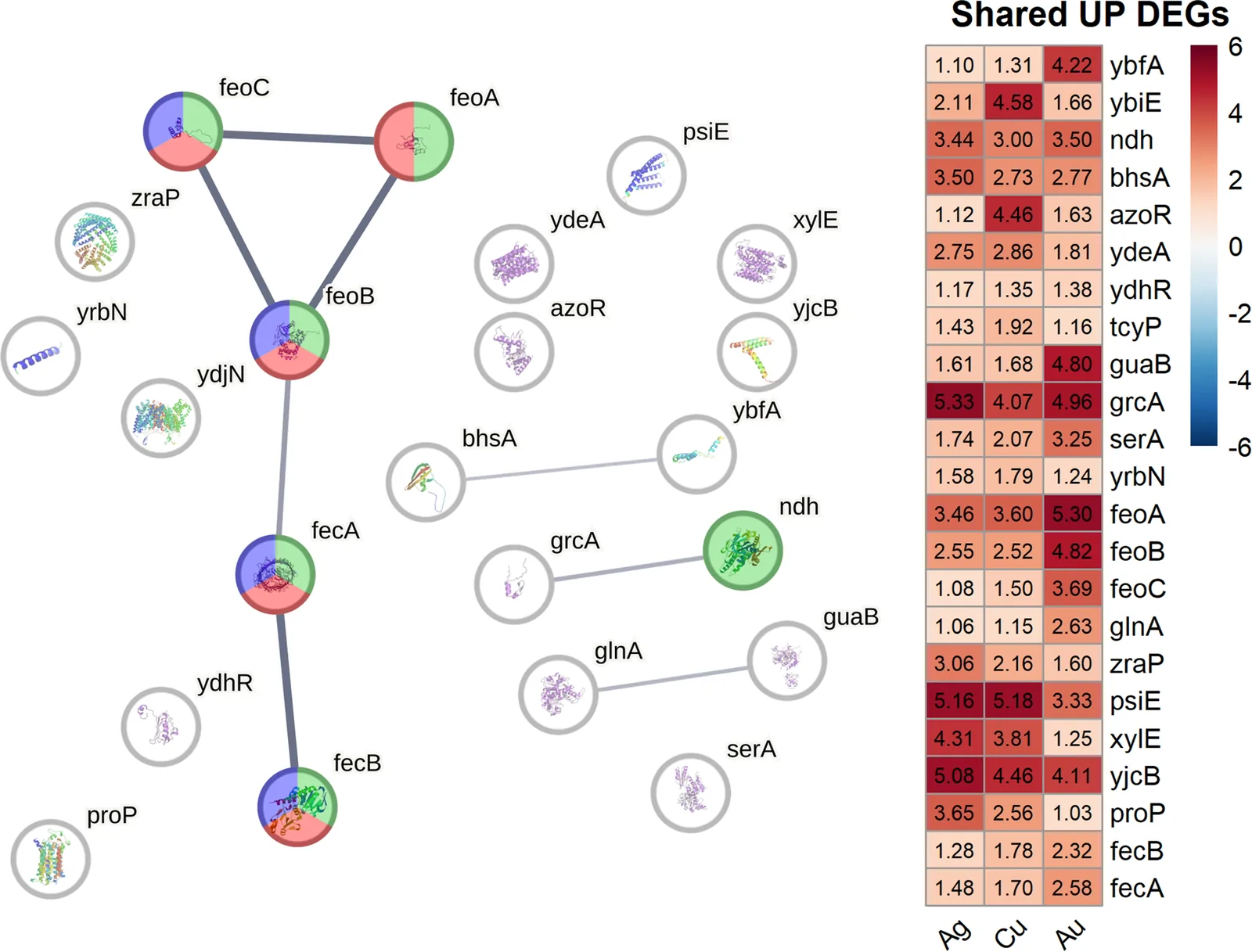
Network interactions map (left) and average log_2_-fold change in gene expression for the 23 shared significant up-regulated genes between the silver nitrate (Ag), copper sulfate (Cu), and tetrachloroauric acid (Au) conditions (right). Each node from the network map represents a gene, and these are connected by edges to adjacent nodes representing a possible interaction between them. A thicker edge between nodes indicates a higher degree of confidence in that interaction. Certain nodes are colored based on the enriched Gene Ontology term they are a part of: green for transition metal ion homeostasis (GO: 0055076), blue for iron import into the cell (GO: 0033212), and red for iron ion homeostasis (GO: 0055072). Each metal salt treatment was contrasted against a non-metal challenge control, with three biological trials each.

Based on the 23 UP DEGs that were shared between the three metal salt treatments, we interrogated the Gene Ontology (GO) enrichments of this subset using the Search Tool for the Retrieval of Interacting Genes/Proteins (STRING) (41). This resulted in three Biological Process GO terms being enriched: transition metal ion homeostasis (GO: 0055076), iron import into the cell (GO: 0033212), and iron ion homeostasis (GO: 0055072) (Fig. 4). As such, it is natural to infer a disruption of iron homeostasis and metabolism caused by the three metal stressors in question. This is further supported by the overall increase in gene expression activity within the Fur regulon (ferric uptake regulation, Fig. S11), which features as one of the regulons with the highest activity across all metal treatments (#1 in Ag and Au, #10 in Cu) (Table 2).

**TABLE 2:** Notable *E. coli* K12 BW25113 regulons implicated in gene expression changes after 10 hours of growth in the presence of silver nitrate (Ag), copper sulfate (Cu), or tetrachloroauric acid (Au), obtained using ISMARA^*a*^

Regulator	Description	Ag		Cu		Au	
		Z-score	Rank	Z-score	Rank	Z-score	Rank
ArgP	Modulates transcription of genes related to arginine and lysine metabolism	0.665	16	0.359	25	4.334	7
ArgR	Represses the expression of arginine biosynthesis genes	0.709	12	−0.299	169	3.748	9
CpxR	Responds to misfolded periplasmic and inner membrane proteins	1.897	2	0.435	23	−0.409	109
CueR	Controls the expression of the copA and cueO copper homeostasis genes	0.186	61	1.851	3	6.01	6
CusR	Copper extrusion system CusCFBA	0.305	39	1.986	1	0.123	75
CysB	Activator of sulfur metabolism and cysteine biosynthesis	0.143	76	1.584	4	1.717	35
Fur	Repressor of ferric uptake regulon. Controls expression of genes involved in iron homeostasis	4.748	1	0.962	10	12.253	1
MetJ	Represses the methionine operon	1.082	7	−0.213	152	9.89	2
TrpR	Expression of the tryptophan biosynthesis regulation and transport regulon	0.417	31	0.137	65	7.936	4
TyrR	Modulates the expression of genes involved in aromatic amino acid biosynthesis and transport	0.264	48	−0.357	175	3.625	10
Zur	Zinc ABC transporter uptake system	0.199	59	−0.204	150	6.075	5

^*a*^ The z-score is a representation of differential expression induced in the target genes as a number of *n* standard deviations away from zero, with scores indicating up- (z-score > 0) or down- (z-score < 0) regulation in these genes. From a total of 207 analyzed regulons, each one is ranked from highest to lowest activity within each treatment.

Fur is a transcriptional regulator that can bind a 2-iron, 2-sulfur cluster ([2Fe–2S]). It has been proposed that its main regulatory role is to act as a sensor of ferrous iron [Fe(II)] within the cell (42). When there is an abundance of intracellular Fe(II), [2Fe–2S] centers become more available and trigger a conformational change in Fur (43), enabling its genetic repressor role. Conversely, when Fe(II) is scarce, Fur repression is alleviated, resulting in a higher expression of its target genes. Seven Fur target genes over four transcriptional units stand out as UP DEGs across the three conditions: NADH-quinone dehydrogenase *ndh*, autonomous glycyl radical cofactor *grcA*, ferrous iron transport system *feoABC*, and ferric [Fe(III)] citrate transport components *fecAB*. The presence of these last two iron import operons would indicate that the three metal salts are inducing an iron-deprived state in their respective cultures. This type of genetic response has also been observed with gallium nitrate (44).

While it is a shared trend, it is possible that each coinage metal elicited this type of response through different mechanisms. Ag(I) is known to attack exposed [Fe–S] clusters (45) given its nature as a soft acid, interacting with thiol ligands (46). Copper, being able to switch between its soft acid cuprous [Cu(I)] and borderline acid cupric [Cu(II)] forms under cellular redox potentials (47), has the ability to compete iron out from these clusters. Both interactions are likely to compromise the integrity of Fur’s [2Fe–2S] cluster. On the other hand, tetrachloroauric acid presents gold in its trivalent oxidation state. Being a harder acid, Au(III) wouldn’t exactly fit Ag(I) or Cu(II)’s models, but it’s been reported that it indirectly triggers a major oxidative imbalance in *E. coli*, characterized by an increase in superoxide (11). Such a phenomenon could be explained by a reduction of Au(III) to Au(I) caused by an undetermined cellular redox activity, which would allow it to compete with Cu(I) sites. It’s been suggested that a local production of Au(I) salts could happen in a reductive environment, such as the *E. coli* cell (48), and metal reduction toward their elemental state is one of the bacterial responses to metal stress (49). This could partly explain the indirect disruption of iron homeostasis and metabolism by Au, although the exact mechanism is yet to be elucidated.

### Expression of the Cus and Cue systems is heavily influenced by the presence of copper

The ability of copper to act as a soft or borderline acid by cycling between Cu(I) and Cu(II) grants a wide range of biochemical applications for this metal. The former tends to have more affinity for soft bases (thioethers and thiols), while the latter shows increased affinity for borderline bases (imidazole nitrogen groups, glutamate, aspartate) (50). As such, it acts as a cofactor in metalloproteins with various functionalities, including oxidation–reduction reactions, electron transfer and transport, and denitrification (51). This feature brings forward a dilemma—copper is an essential trace element, but too much copper becomes toxic due to (i) its tendency to displace other essential metals from their ligands (such as zinc and iron) (52, 53), and (ii) its ability to catalyze hydroxyl radical formation through Fenton chemistry (54, 55).

To protect against copper toxicity, microorganisms have evolved mechanisms that directly control its homeostasis. *E. coli* relies on two main systems to achieve this, each regulated separately. The cytoplasmic CueR requires two Cu(I) ions to become active and promote the transcription of *cueO* and *copA* (56), acting as a de facto Cu(I) sensor in the cytoplasm. This triggers a detoxifying event in which the P-type ATPase CopA and its Cu(I) cytoplasmic chaperone CopA(Z) isoform work in tandem to efflux Cu(I) ions to the periplasm (57–60), where they are oxidized to the less toxic Cu(II) form by CueO (61). On the other hand, the two-component signal transduction system CusSR senses increases in the periplasmic concentration of Cu(I) and promotes the transcription of the Cus system (56, 62), comprised of the *cusCFBA* and *cusSR* transcriptional units. The RND efflux complex CusABC extrudes Cu(I) ions accumulated in the periplasm and cytoplasm (63). It is assisted by the Cu(I) chaperone CusF, which works in tandem with CopA to transfer heavy metal ions to CusABC (64).

**Fig 5:**
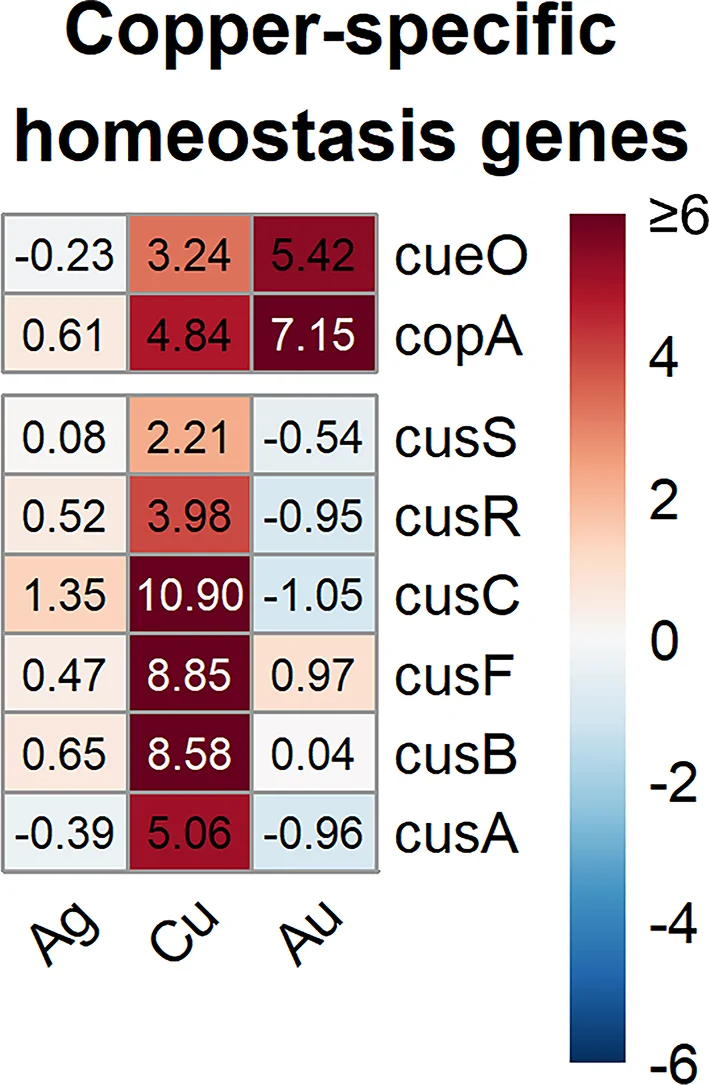
Average log_2_-fold change in gene expression for genes that are known to play a central role in maintaining copper homeostasis in *E. coli* K12 BW25113. The first pair of genes is regulated by CueR, while the second cluster is regulated by CusR. Each column corresponds to a different experimental condition: silver nitrate (Ag), copper sulfate (Cu), and tetrachloroauric acid (Au). Every experiment was contrasted against a non-metal challenge control, with three biological trials each.

In our experiment, Cu stress caused a notable increase in gene expression for both copper homeostasis genes in the CueR regulon, as expected (Table 3). Surprisingly, Au presented an even sharper increase, while Ag only increased *copA* expression marginally (Fig. 5). Meanwhile, the CusR regulon was significantly up-regulated by Cu, with only CusC being an UP DEG in the Ag treatment.

**TABLE 3:** List of atomic and chemical properties from each coinage metal

Property		Copper	Silver	Gold	Reference
Atomic number; AN		29	47	79	(65)
Atomic weight; AW		63.546	107.868	196.967	(65)
Electronic configuration		[Ar] 3d^10^ 4s^1^	[Kr] 4d^10^ 5s^1^	[Xe] 4f^14^ 5d^10^ 6s^1^	(65)
Electronegativity; X_m_		1.65	1.93	2.54	(38)
Ionization energy, by orbital (kJ/mol)	1st	745.3	730.8	889.9	(65)
	2nd	1,957.3	2,072.6	1,973.3	
	3rd	3,577.6	3,359.4	2,895	
Atomic radius; AR (pm)		128	144	144	(65)
Redox potential; ∆E (V)		+0.15 Cu(II)/Cu(s)	+0.79 Ag(I)/Ag(s)	+1.50 Au(III)/Au(s)	(66)
Enthalpies of atomization (kJ/mol)		337	284	379	(65)
Metal sulfide affinity; pK_sp_		40.3	53.6	72.8	(38)
Electron density; log(AR/AW)		−1.672	−1.851	−2.136	(38)
Pearson’s softness index; σ_p_		0.104	0.74	0.44	(38)
Covalent index; X_m_^2^AR		1.987	4.283	5.483	(38)

There is evidence in the literature of CueR responding to the presence of Ag, Cu, and Au metal salts within 3 hours of exposure (37, 48, 67), even if the copper-detoxifying functions of CueO or CopA are not necessarily compatible with silver (68) or gold (67), respectively. This is notable because CueR is reported to have a high selectivity for monovalent metal cations (37), which would fit with the possibility of a localized production of Au(I) salts within the reductive environment of an *E. coli* cell (48). On the other hand, the increase in gene expression of the CusR regulon caused by Cu is a validation of copper efflux being one of the essential mechanisms to cope with an excess of this metal. While it’s been reported in other short-term exposure studies that the Cus system can also respond to Ag(I) ions (69) and efflux them (70), the increase in expression activity by the Ag treatment was markedly smaller than that of Cu exposure. Beyond their known role in maintaining copper homeostasis, our results add nuance to the function of the Cue and Cus systems after acclimation to Ag and Au stress, and how the induction of these systems by Ag decreases after initial exposure.

### Silver activates the Cpx cell envelope stress response

One of the unique signals we detected in our Ag experiment was the increased gene expression activity in the CpxR regulon. The Cpx response is composed of a two-component signal transduction system (the sensor kinase CpxA and the transcriptional regulator CpxR), and senses misfolding and misplacement of proteins in the cell envelope (71). Multiple stimuli have been documented to induce it, including acute exposure to copper chloride (CuCl_2_) (72, 73). A model has been proposed where Cu(I) inhibits lipoprotein maturation while they are in traffic from the inner membrane (IM) to the outer membrane (OM). This prevents the sentry lipoprotein NlpE from reaching the OM, causing it to accumulate in the IM, which ultimately triggers the Cpx response (36).

**Fig 6:**
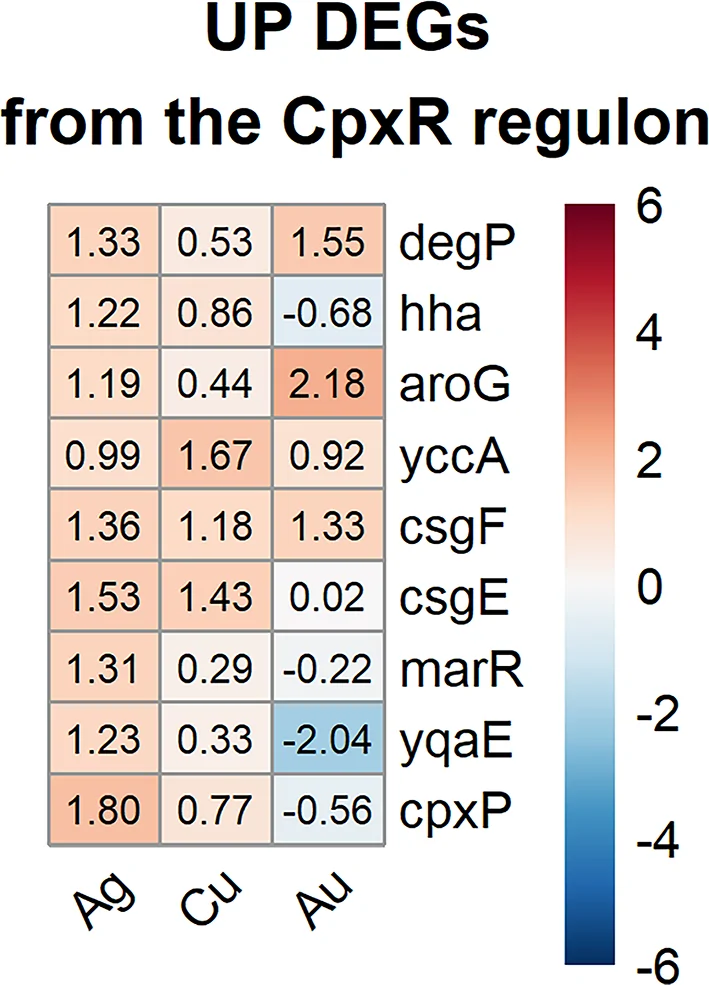
Average log_2_-fold change in gene expression for CpxR-regulated differentially expressed genes for the Ag and Cu treatments in *E. coli*. Each column corresponds to a different experimental condition: silver nitrate (Ag), copper sulfate (Cu), and tetrachloroauric acid (Au). Every experiment was contrasted against a non-metal challenge control, with three biological trials each.

While silver nitrate toxicity is known to attack the cell envelope (74), we believe this is the first report of an up-regulation of the CpxR regulon by this metal stressor. Eight CpxR-regulated genes were above significance thresholds in our Ag experiment: periplasmic serine endoprotease *degP*, hemolysin expression modulator *hha*, 3-deoxy-7-phosphoheptulonate synthase *aroG*, curli assembly components *csgFE*, multiple antibiotic resistance transcriptional regulator *marR*, predicted inner membrane protein *yqaE*, and Cpx response modulator *cpxP*. All of these demonstrated slight increases in expression in the Cu data set, with *csgFE* and FtsH protease modulator *yccA* being UP DEGs as well (Fig. 6). Considering that both Cu(I) and Ag(I) ions (soft acids) will tend to attack thiol groups from cysteine residues (soft base) (46), it is theoretically possible for both to oxidize thiols to induce this type of response. Nonetheless, this induction of the Cpx response from Ag and Cu supports cell envelope homeostasis as a target for silver ion toxicity. This is consistent with shorter-term observations of induction of CpxR-related genes in previous studies, as this regulon has been shown to respond to Cu stress after 30 minutes of exposure (73), and the *cpxR* gene itself has been reported to be up-regulated more than twofold after 10 minutes of silver nanoparticle exposure (75).

### Genes related to reduced thiol and redox balance are induced by copper and gold

Metal stress has been shown to cause a spike in oxidative stress upon exposure (76), which results in redox imbalance and a depletion of the antioxidant pool of the cell. When looking at our data sets, none showed a significant increase in expression activity for any of the regulons mainly implicated in the immediate response to oxidative stress—OxyR and SoxRS (Table S7). This is an expected outcome considering that these regulons are more active upon peak oxidative stress shock, as *soxS* has been observed to be up-regulated >600-fold 10 minutes after silver chloride exposure (75), but their regulon-wide activity winds down once redox balance has been reestablished (77), which is consistent with the acclimation model from our experiment.

**Fig 7:**
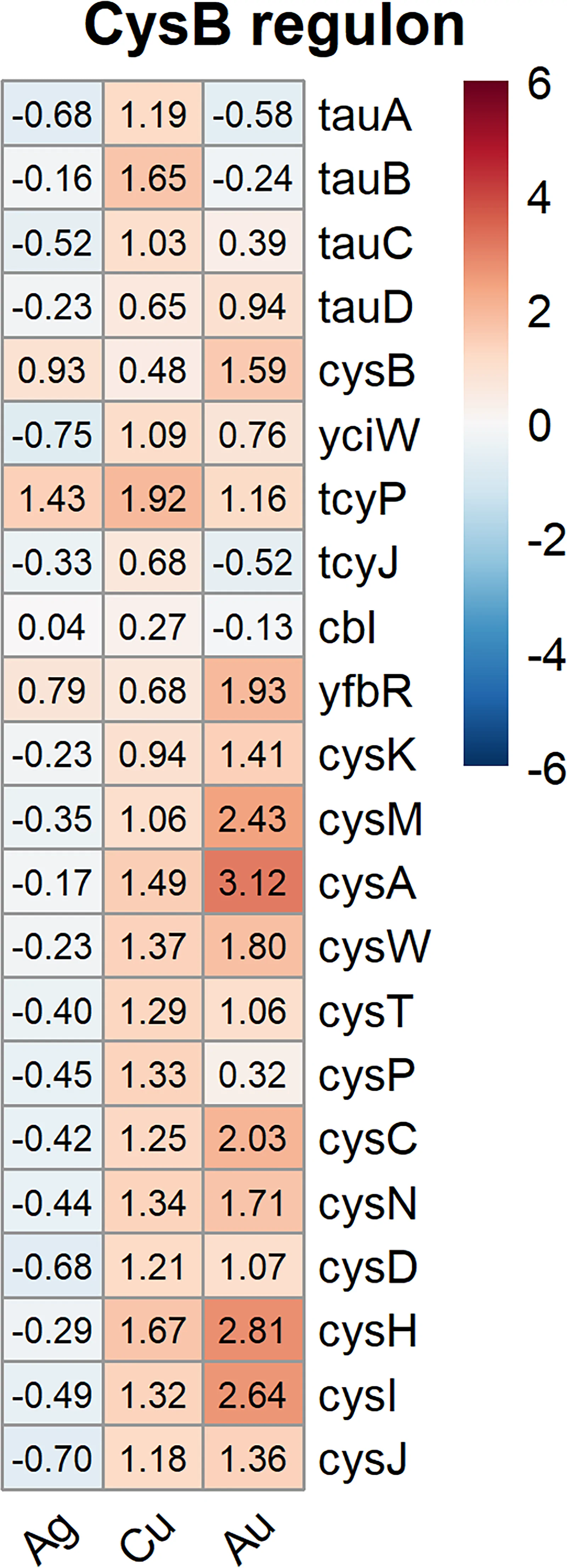
Average log_2_-fold change in gene expression for CysB-regulated genes in *E. coli* K12 BW25113. Each column corresponds to a different experimental condition: silver nitrate (Ag), copper sulfate (Cu), and tetrachloroauric acid (Au). Every experiment was contrasted against a non-metal challenge control, with three biological trials each.

One of the consequences of these spikes in oxidative stress is an increase in reduced thiols due to the antioxidant pool of the cell being depleted (11). For this reason, we decided to monitor the CysB regulon, which had high expression activities in the Cu and Au data sets (Fig. 7). This transcription factor regulates genes involved in pathways, such as cysteine biosynthesis and sulfate assimilation, having a central role in replenishing the pools of antioxidant molecules, such as glutathione and thioredoxin (78). Both Cu and Au show an overall trend of up-regulation, with Cu having 16 out of 22 genes being transcribed more than twofold, and Au having the highest expression numbers. This is compounded by the up-regulation of (i) *trxB* (log_2_-fold change: Au 2.25), which codes for thioredoxin reductase and is a known target for auranofin toxicity (79), and (ii) the *gsiABCD* transcriptional unit (average log_2_-fold change: Cu 1.18, Au 1.44) that codes for a glutathione import system. The latter is not part of the CysB regulon but is often co-expressed with some of those genes as part of the Cysteine-1 iModulon (32), which was up-regulated by Au (Fig. 5).

While these two metals reported high expression activities in the CysB regulon, Ag did not elicit the same type of response, having most of this regulon down-regulated. Despite this contrast, the CysB-regulated *tcyP* appeared as an UP DEG across our three metal treatments. This gene codes for a L-cystine/L-cysteine shuttle transport system, which provides reducing equivalents to the periplasm, helping restore redox balance as part of the *E. coli* response to oxidative stress (80). Along with the CysB regulon, *tcyP* was also significantly up-regulated in a previous acclimation experiment using gallium nitrate (44). These observations at large suggest that both the CysB regulon and the TcyP exchanger could play an important role in protecting against oxidative stress induced by toxic metals over longer exposure times by helping cells maintain a steady supply of antioxidant molecules. Considering that genes from the Cys family have been reported to be up-regulated within 30 minutes of silver and copper exposure (73, 75), this also reflects how the adaptive response to each metal differs over time, as they seem to retain high expression levels after acclimation is reached for Cu but not for Ag.

### Gold affects the expression of genes related to protein translation, ribosome biogenesis, and zinc uptake

Our Au data set is the first published data set that explores the transcriptome-wide response to Au(III) after 10 hours of growth. This experiment yielded the transcriptional profile that differed the most from its non-metal-treated control, with 46.89% of our strain’s coding sequences being differentially expressed. Part of this contrast can be explained by what seems to be an increase in protein synthesis. The Translation iModulon (Fig. 2) presents the highest activity in our Au treatment. This cluster of co-expressed genes is mostly composed of ribosomal subunit proteins related to translation or ribosomal structure and biogenesis (32). One of the common transcription factors of 44 out of 53 genes from this group is DksA, an autoregulated DOWN DEG (log_2_-fold change: −1.12) which controls the expression of a variety of operons related to amino acid biosynthesis and ribosomal subunits. Furthermore, the up-regulation of several amino acid biosynthesis regulons (MetJ, TrpR, ArgP, ArgR, TyrR; Table S3) and iModulons (Histidine, Tyr/Trp/Phe, Tryptophan, Leu/Val/Ile, Cysteine-2; Fig. 2) suggests an increased demand of amino acids for translation. This evidence leads us to believe that Au exposure is somehow leading to protein breakdown or ribosome disassembly at a greater degree than Ag and Cu, eliciting this kind of response. While direct evidence of gold ion toxicity targeting ribosomes is limited, there is precedent of gold nanoparticles affecting ribosome-tRNA binding by another study (81). It is reasonable to assume a possible path of toxicity by means of interactions between gold ions with exposed thiol and thioether groups from ribosomal proteins, similar to previous observations with auranofin (82).

**Fig 8:**
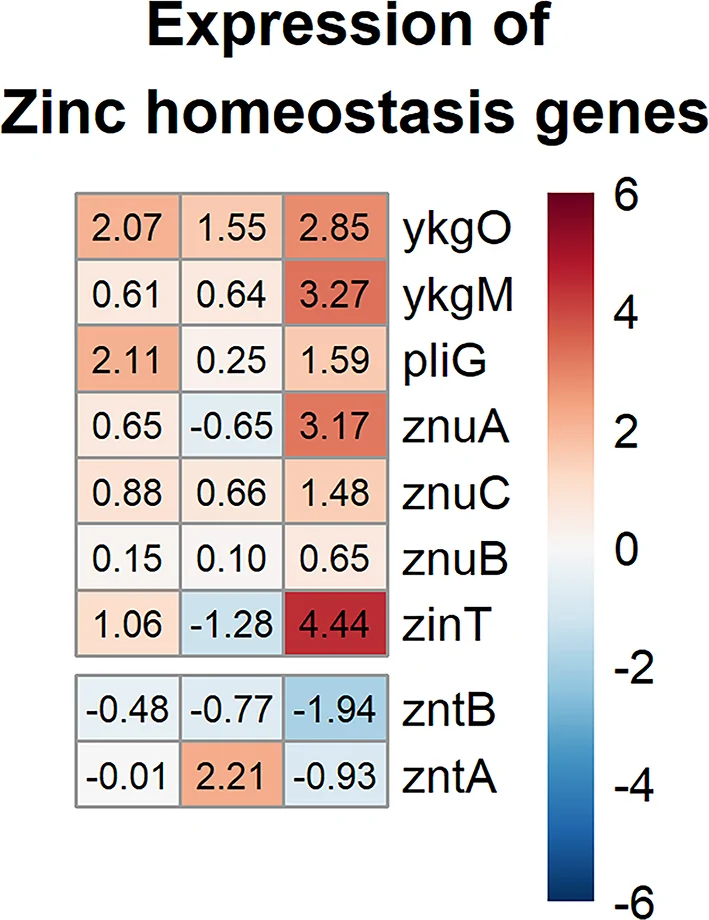
Average log_2_-fold change in gene expression for genes that are known to play a central role in maintaining zinc homeostasis in *E. coli* K12 BW25113. The first group of genes is regulated by Zur, while the lower pair is regulated by ZntR. Each column corresponds to a different experimental condition: silver nitrate (Ag), copper sulfate (Cu), and tetrachloroauric acid (Au). Every experiment was contrasted against a non-metal challenge control, with three biological trials each.

Another regulon that stood out from our Au experiment is the one controlled by the zinc uptake regulator Zur. This transcription factor uses four Zn(II) atoms in its active DNA-binding form, repressing the transcription of its target genes (83), including the zinc uptake system ZnuABC and its chaperone ZinT. All of Zur’s target genes are up-regulated in our Au condition, with only one just below significance levels (Fig. 8). This observation is consistent with the down-regulation of z*ntA*, which encodes for a P-type ATPase that effluxes Zn(II) ions from the cytoplasm to the periplasm (84). *zntA* transcription is activated by ZntR in the presence of Zn(II), and its activation is also dependent on the availability of these ions. Therefore, this could be an indication that our Au treatment may be leading to a zinc-deprived state, commanding a higher demand for Zn(II).

### Concluding remarks

For decades, the longstanding dogma was that metal toxicity in bacteria consisted of increased oxidative stress that ultimately results in bacterial death. This is still seen in some studies submitted recently, and it has led to the misconception that (i) all metals behave in the exact same way, and (ii) bacteria respond to all of them similarly. Nonetheless, an early study highlighted considerable differences between the metal ions’ oxidation response in *E. coli* (76). It is now known that metal elements interact with the cellular biochemistry in different and complex ways in accordance with their physicochemical properties (6, 50) and the physiological state of the cells. Using a transcriptomic approach, our study now adds additional information on how different the responses can be. We provided a model that depicts shared and unique traits in the *E. coli* adaptive and intrinsic gene expression response to each antimicrobial coinage metal at sublethal concentrations. Crucially, we identify changes in bacterial physiology of cultures that have acclimated to sublethal inhibitory metal stress, giving clues into what type of response is needed for sustained growth in these conditions. Disruption in iron metabolism is to be expected as a shared response since [Fe–S] centers are prime targets for coinage metal toxicity, promoting activity in the Fur regulon. Activation of the copper homeostasis system Cus and Cue by Cu was also expected, although it is interesting that Au only activated the latter, while both were relatively unresponsive to Ag. Induction of the Cpx response is also consistent with previous observations of Ag toxicity attacking the cell envelope. The stark difference in transcriptomic profile elicited by Au was a surprise, majorly characterized by increased gene expression in sulfur metabolism pathways (controlled by CysB, also elicited by Cu), translation and ribosome biogenesis genes, and the zinc uptake regulon Zur (Fig. 7). Compared to transcriptome-wide data sets in the literature that focus on the cellular response within 3 hours of exposure to each metal, our data sets suggest a deviation away from the initial SOS response (characterized by upregulation of oxidative stress and efflux-related genes) toward the transcriptional trends previously described by each metal. These observations fill knowledge gaps of metal-bacteria interactions, going beyond the standard acute toxicity models to gain a better understanding of bacterial physiology adapted to grow in the presence of these antimicrobial metals.

## MATERIALS AND METHODS

### Sublethal concentration determination

*E. coli* K12 BW25113 stored at −70°C was thawed and cultured in Lysogeny Broth media (10 g/L tryptone, 10 g/L NaCl, 5 g/L yeast extract) overnight at 37°C, 150 rpm in a shaker incubator to recover the bacterial strain. For each experiment in a 250 mL Erlenmeyer flask, 500 µL of a bacterial overnight culture was used to inoculate 7.5 mL of 2× M9-glucose minimal media diluted to a 1× concentration (6.8 g/L Na_2_HPO_4_, 3 g/L KH_2_PO_4_, 1 g/L NH_4_Cl, 0.5 g/L NaCl, 4 mg/L glucose, 0.5 mg/L MgSO_4_, and 0.1 mg/L CaCl_2_) with 7.5 mL of metal salt (AgNO_3_, CuSO_4_, or HAuCl_4_) in aqueous solution. Untreated positive controls for bacterial growth used sterile deionized water [(dd)H_2_O] instead of the metal salt component, while negative controls for media sterility had culture media and (dd)H_2_O but no inoculum.

Each flask was incubated in a shaker incubator at 37°C, 150 rpm. Optical density at 600 nm (OD600) was determined using a spectrophotometer. Relative to the untreated control, possible sublethal inhibitory concentrations were determined to have either (i) an extended lag phase that suggests an adjustment of the bacterial physiology to the metal-spiked media, (ii) a less steep slope during the exponential growth phase indicating reduced doubling times, or (iii) a reduced OD600 value (between 45% and 95%) compared to the OD600 from the untreated control after 10 hours of growth defining inability to reach saturating cell numbers.

### Total RNA sample preparation

Experimental treatments of AgNO_3_ 7 µM, CuSO_4_ 39 µM, or HAuCl_4_ 10 µM were set up along with their respective non-metal controls as described above. Ten hours after inoculation, 2 mL of bacterial culture was centrifuged at 14,000 × g for 2 minutes to pellet the cells. Total RNA was then extracted using RiboPure-Bacteria RNA Isolation Kit AM1925 (Invitrogen, USA) according to the manufacturer’s recommendations, including DNase treatment. Three biological trials were set up for both conditions.

### RNA sequencing

Samples were sent to the Center for Health Genomics and Informatics and the UCDNA Sequencing facility (Cumming School of Medicine, University of Calgary, Canada) for sequencing. Ribosomal RNA depletion was performed using the NEB rRNA Depletion Bacterial Module (New England Biolabs, USA). The cDNA library preparation for Illumina sequencing was executed using the NEBNext Ultra II Directional RNA Library Prep Kit and NEB Indexing Kit (New England Biolabs, USA). After adaptor ligation and amplification, the average library fragment size (in base pairs) for each data set was as follows: Ag 371, Cu 366, and Au 362. Libraries were quantified using the KAPA qPCR Library Quantification Kit for Illumina platforms, then pooled and loaded on the Illumina MiSeq sequencer at a concentration of 12 pM. The libraries were sequenced using a 2 × 75 bp 150-cycle run, with a total output of 25 M read pairs.

### Differentially expressed genes identification, quantification, and analysis

Sequencing files quality control, quantification of gene expression, and DEG analysis were performed in a variety of R-Studio library scripts. Initial FASTQ files (submitted to NCBI Sequence Read Archive under BioProject accession numbers PRJNA1256886—Copper, PRJNA1256887—Silver, PRJNA1256888—Gold) were assessed and trimmed with the Bioconductor package *rfastp* (85) (version 1.10). Gene expression was assessed by aligning to the *E. coli* K12 BW25113 RefSeq assembly annotation file GCF_000750555.1 using the Bioconductor package *Rsubread* (86) (version 2.14.2), with counts assigned to the CDS meta-feature (Tables S1–S3). Finally, the counts matrix for the 4,378 identified features was imported into the Bioconductor package *DESeq2* (87) (version 1.40.2) for DEG determination. *P* values were calculated using the Wald test. The adjusted *P* value (*P*-adj) for multiple test adjustment (Benjamini-Hochberg correction, FDR < 0.05) was set to 0.05. DEGs were defined as those with a *P*-adj value < 0.05 and whose expression had an absolute fold-change (FC) of at least 2 (|log_2_ FC| > 1) when contrasting each metal salt treatment versus their respective unchallenged controls; those with a negative FC were deemed as down-regulated genes in each metal salt treatment, while a positive FC indicated up-regulation (Tables S1 through S3). Functional enrichment of individual genes was further annotated through the UniProt and EcoCyc databases. Functional network enrichments were performed by submitting lists of genes of interest to the STRING web software (41).

The ISMARA web software (Swiss Institute of Bioinformatics, Basel, Switzerland) (31) was used to detect motifs in the promoters of the *E. coli* K12 genome and infer the activity of gene regulators (including transcription factors, small RNA, and RNA-polymerase subunits) based on the expression of their target genes. Our FASTQ files were uploaded to the ISMARA web server and averaged together by treatment. The full list of results is included in Table S7.

For our co-expression analysis, the *E. coli* PRECISE-1K data set (33) was utilized, and the activity of each of the 201 iModulons was determined for both conditions of our experiment, as indicated by Lamoreux et al. The difference in activity between both conditions represents differential iModulon activity between our treatments, with positive values indicating higher activity in each metal salt treatment, and negative ones representing lower activity. Statistical significance was calculated using the cumulative log-normal distribution of each iModulon across all the conditions.

## Data Availability

Processed transcriptomic data (sequencing files, raw counts matrix, and *DESeq2* output table) are available under NCBI Gene Expression Omnibus accession numbers GSE295970 (copper), GSE295971 (gold), and GSE295972 (silver).

## SUPPLEMENTAL MATERIAL

Supplemental figures and tables spectrum.02541-25-s0001.docx Figures S1 to S11 and Tables S1 to S3.

Tables S4 to S7 spectrum.02541-25-s0002.xlsx Gene counts, differential expression analysis, and functional annotation of our datasets.

ASM does not own the copyrights to Supplemental Material that may be linked to, or accessed through, an article. The authors have granted ASM a non-exclusive, world-wide license to publish the Supplemental Material files. Please contact the corresponding author directly for reuse.
