# Supplementary material for: Contrasts and similarities in the transcriptomic response to antimicrobial coinage metals in Escherichia coli
Source: Microbiol Spectr. 2026 Jun 15;14(7):e02541-25. doi: 10.1128/spectrum.02541-25 (PMC13339815; doi:10.1128/spectrum.02541-25)
Supplement: Supplemental figures and tables — Figures S1 to S11 and Tables S1 to S3. [file spectrum.02541-25-s0001.docx]

**SUPPLEMENTARY FIGURES & TABLES**

**Contrasts and similarities in the transcriptomic response
to antimicrobial coinage metals in *Escherichia coli***

Daniel A. Salazar-Alemán, Ashley McGibbon, and Raymond J. Turner

**
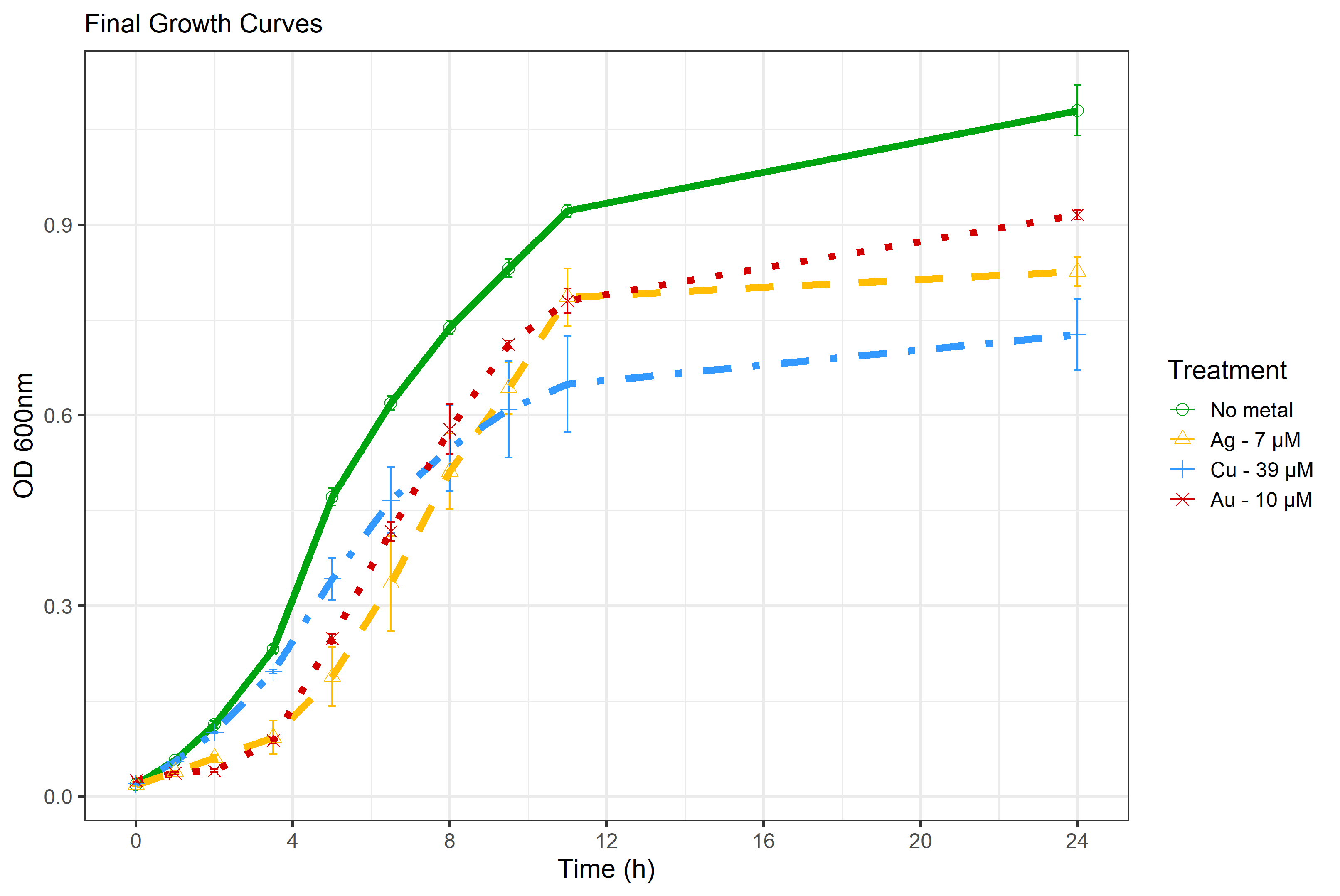
**

**Figure S1**. Growth of *E. coli* K12 BW25113 in the presence of sublethal inhibitory concentrations of silver nitrate (Ag), copper sulfate (Cu) and tetrachloroauric acid (Au). Cells were incubated in 250 mL Erlenmeyer flasks at 37 ºC 150 rpm using 15 mL of M9-glucose minimal media, spiked with their respective metal salt. Each symbol point is the average of three biological trials. Error bars represent one standard deviation.


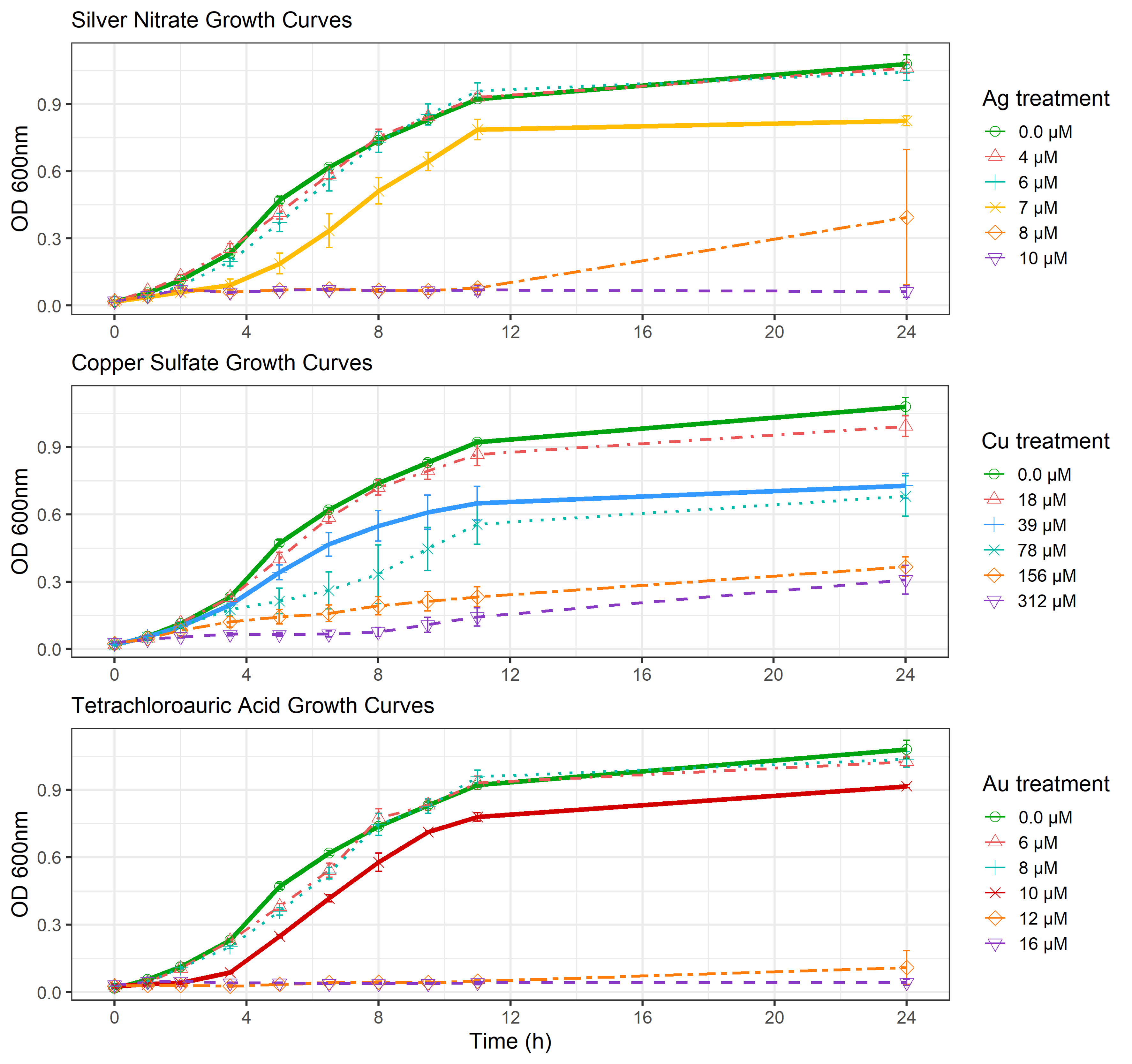


**Figures S2-S4**. Growth of *E. coli* K12 BW25113 in the presence of coinage metal salts. Cells were incubated in 250 mL Erlenmeyer flasks at 37 ºC 150 rpm using 15 mL of M9-glucose minimal media, spiked with their respective metal salt: silver nitrate (Ag, top), copper sulfate (Cu, middle), and tetrachloroauric acid (Au, bottom). Each symbol point is the average of three biological trials. Error bars represent one standard deviation.

**
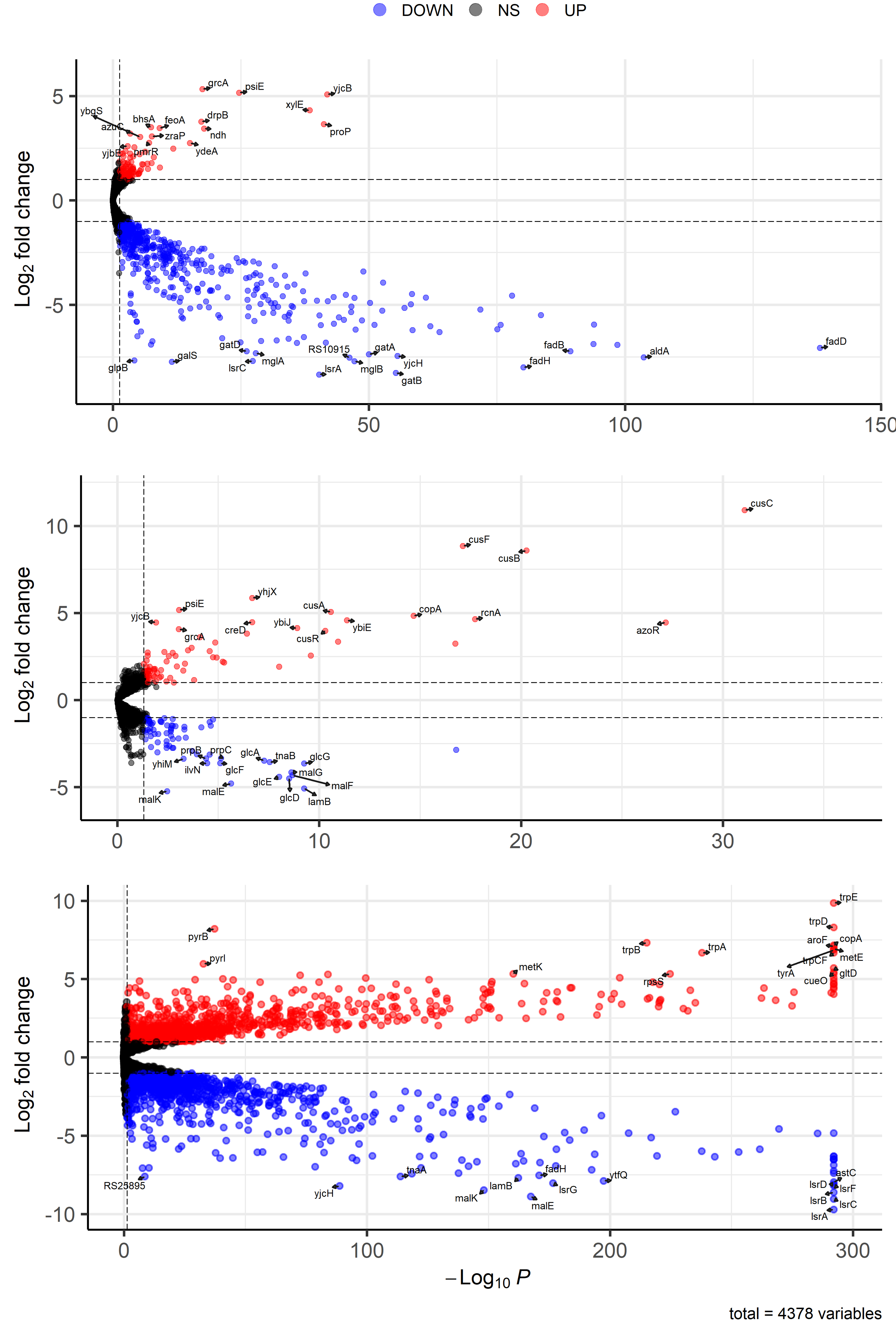
**

**Figures S5-S7**. Volcano plots showing differential gene expression of *E. coli* K12 BW25113 after growing in sublethal concentrations of silver nitrate- (top), copper sulfate- (middle) and tetrachloroauric acid- (bottom) spiked M9-glucose minimal media for 10 hours, contrasted against an untreated control. The thresholds for significance were defined by false discovery rate-adjusted p-value < 0.05 and absolute fold-change ≥ 2 (|log_2_ fold change| ≥ 1). Up-regulated differentially expressed genes (DEGs) that exceeded these thresholds are pictured with a red dot, down-regulated DEGs have a blue one; labels with the gene name appear for the top 15 up- and down-regulated DEGs.

**
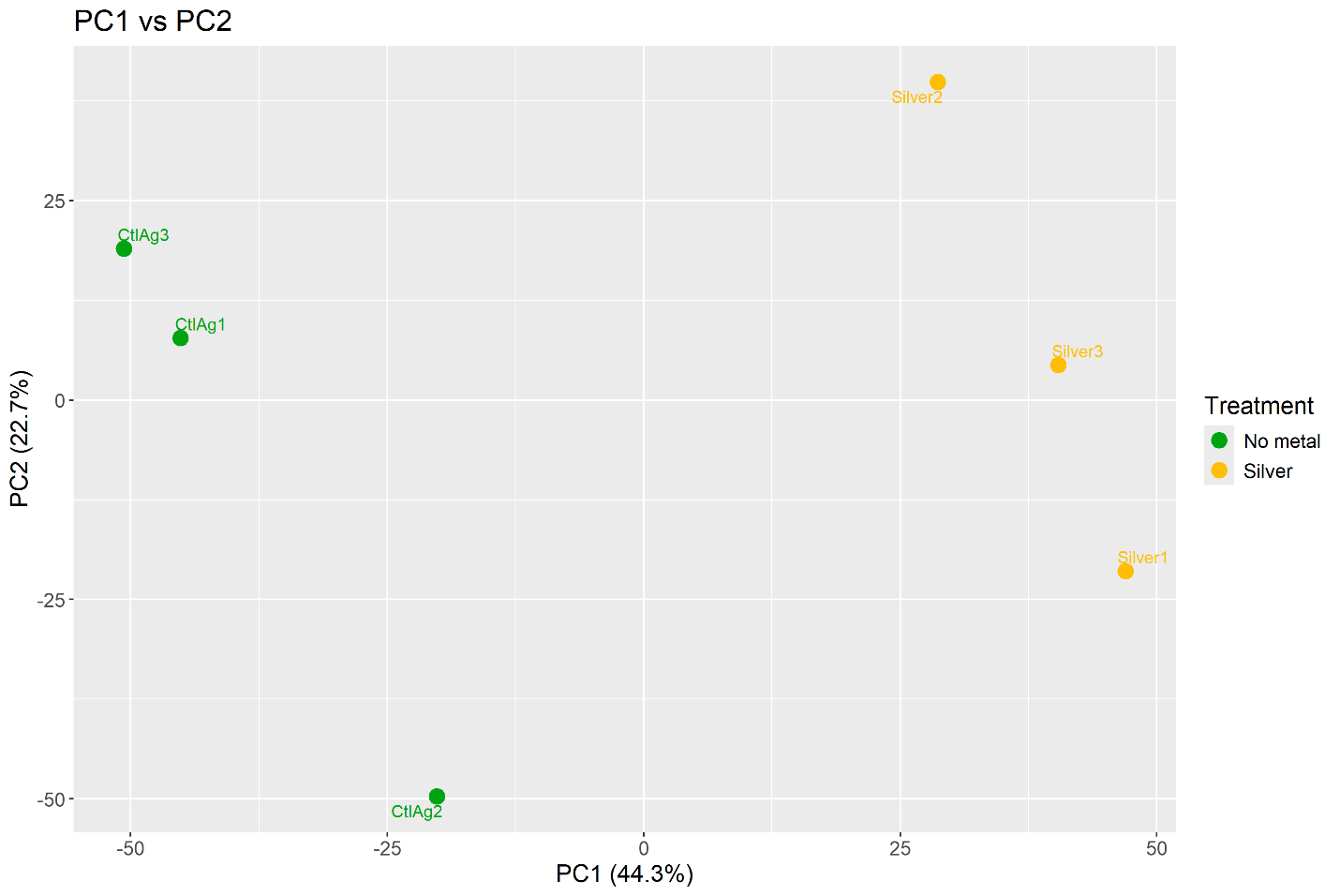
**

**Figure S8**. Principal Component Analysis of the samples from the silver nitrate RNA-seq experiment. The r-log transformed raw count values of the 219 most variable genes across samples were used, representing 5% of the 4378 total genes in *E. coli* K12 BW25113. The distances between each point characterize the variation in expression between each sample.

**
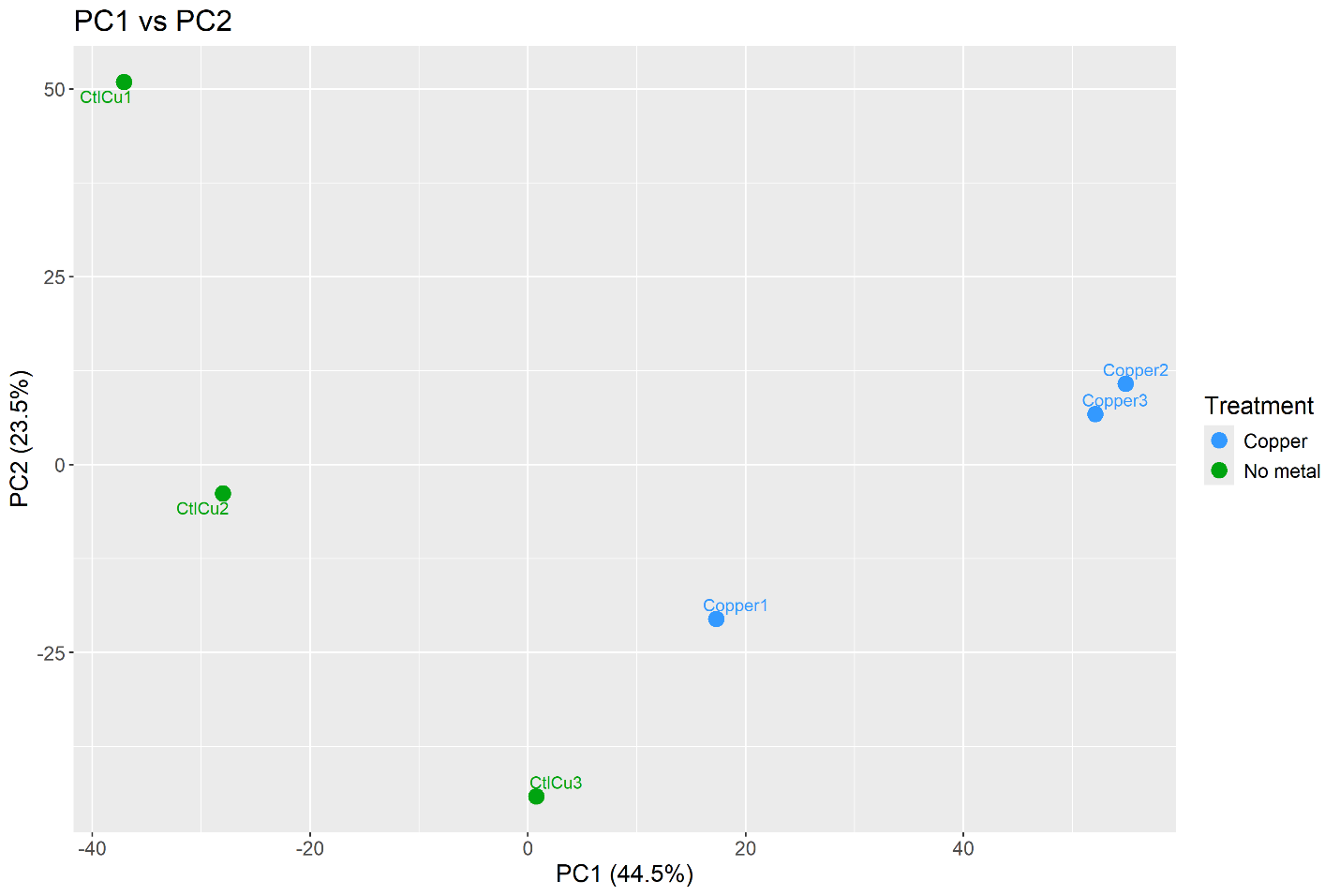
**

**Figure S9**. Principal Component Analysis of the samples from the copper sulfate RNA-seq experiment. The r-log transformed raw count values of the 219 most variable genes across samples were used, representing 5% of the 4378 total genes in *E. coli* K12 BW25113. The distances between each point characterize the variation in expression between each sample.

**
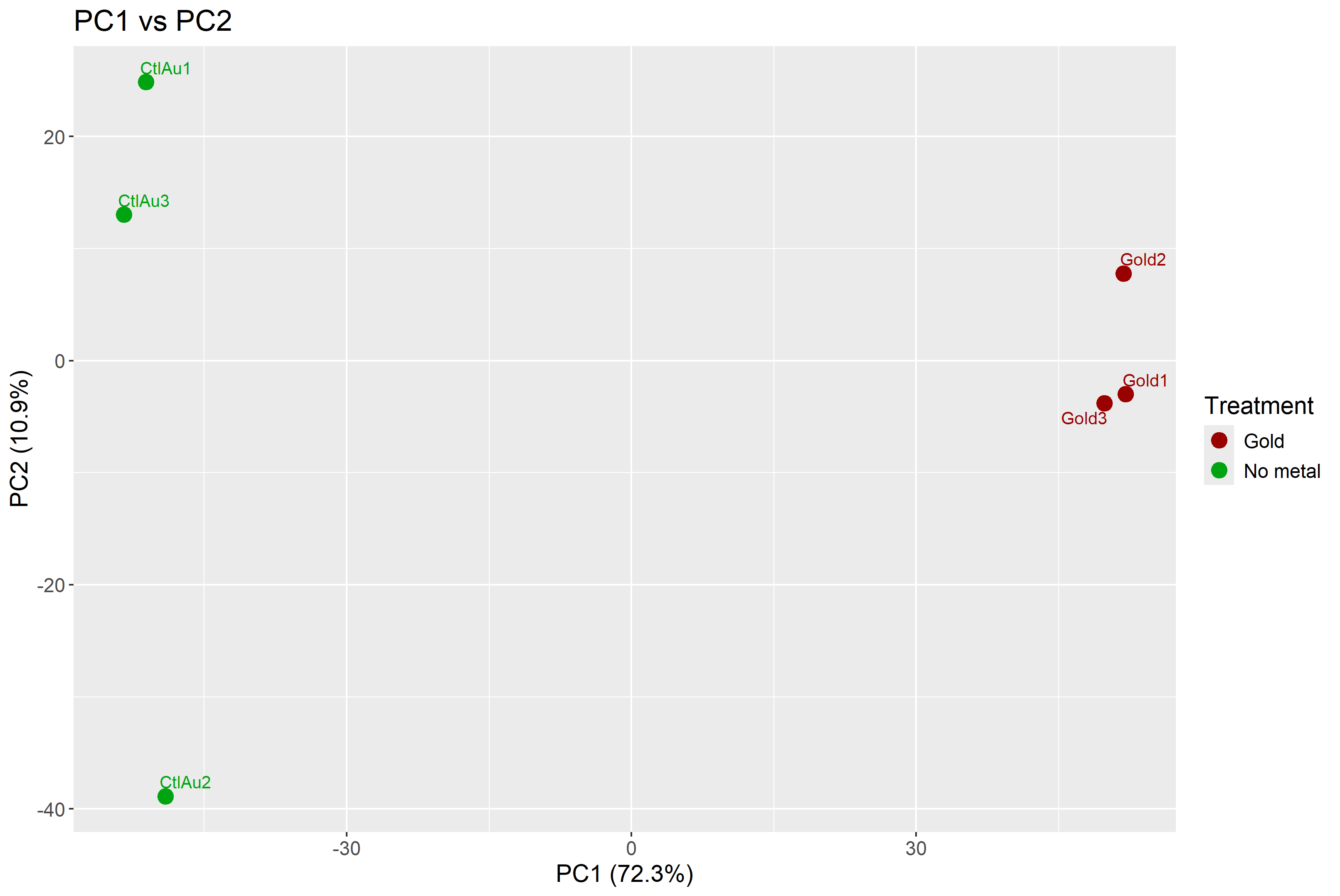
**

**Figure S10**. Principal Component Analysis of the samples from the tetrachloroauric acid RNA-seq experiment. The r-log transformed raw count values of the 219 most variable genes across samples were used, representing 5% of the 4378 total genes in *E. coli* K12 BW25113. The distances between each point characterize the variation in expression between each sample.

**Supplementary Table 1**. The top and bottom 10 *E. coli* K12 BW25113 regulators implicated in positive gene expression changes after 10 hours of growth in the presence of sublethal concentrations of silver nitrate, obtained using ISMARA. The z-score is a representation of differential expression induced in the target genes as a number of *n* standard deviations away from zero, with scores indicating up- (z-score > 0) or down- (z-score < 0) regulation in these genes.

| **Regulator** | **Description** | **Z-score** |
| --- | --- | --- |
| Fur | Repressor of ferric uptake regulon. Controls expression of genes involved in iron homeostasis. | **4.748** |
| CpxR | Responds to misfolded periplasmic and inner membrane proteins. | **1.897** |
| Sigma70 | RNA polymerase, sigma D factor. Genes associated with fast cellular growth. | **1.758** |
| PdhR | Activation of fatty acid degradation genes and repression of cell mobility genes. | **1.533** |
| NagC | Coordinates the biosynthesis of D-glucosamine and N-acetylglucosamine with their catabolism. | **1.318** |
| IHF | DNA-bending protein that affects transcription of a wide variety of operons. | **1.123** |
| MetJ | Represses the methionine operon. | **1.082** |
| PhoP | Regulates genes involved in magnesium uptake, acid resistance and lipopolysaccharide modification. | **1.054** |
| GadE | Central activator of the acid response system. | **0.916** |
| Cra | Helps modulate the flow of central carbon metabolism. | **0.854** |
| GalR | Repressor of transport and catabolism of D-galactose. | **-1.197** |
| MalT | Activator of the maltose regulon: uptake and catabolism of malto-oligosaccharides. | **-1.214** |
| UxuR | Repressor of transport and catabolism of β-D-glucuronides, glucuronate, and gluconate. | **-1.227** |
| GlpR | Repressor of transport and catabolism of glycerol-3-phosphate. | **-1.302** |
| Sigma54 | RNA polymerase, sigma N factor. Genes associated with nitrogen-related processes. | **-1.340** |
| TorR | Activates the trimethilamine-N-oxide anaerobic respiratory system. | **-1.359** |
| FlhDC | Regulates flagella formation, operation, and carbon source metabolism. | **-1.371** |
| FadR | Regulates fatty acid degradation while it activates fatty acid biosynthesis. | **-3.648** |
| ArcA | Repressor of a wide variety of aerobic enzymes under anaerobic conditions. | **-4.982** |
| CRP | Global activator of secondary carbon source pathways. | **-13.487** |

**Supplementary Table 2**. The top and bottom 10 *E. coli* K12 BW25113 regulators implicated in positive gene expression changes after 10 hours of growth in the presence of sublethal concentrations of copper sulfate, obtained using ISMARA. The z-score is a representation of differential expression induced in the target genes as a number of *n* standard deviations away from zero, with scores indicating up- (z-score > 0) or down- (z-score < 0) regulation in these genes.

| **Regulator** | **Description** | **Z-score** |
| --- | --- | --- |
| CusR | Copper extrusion system CusCFBA. | **1.986** |
| YedW | Hydrogen peroxide response regulator. | **1.986** |
| CueR | Controls the expression of the *copA* and *cueO* copper homeostasis genes. | **1.851** |
| CysB | Activator of sulfur metabolism and cysteine biosynthesis. | **1.584** |
| PhoB | Activates expression of the *pho* regulon in response to environmental inorganic phosphate. | **1.398** |
| Sigma70 | RNA polymerase, sigma D factor. Genes associated with fast cellular growth. | **1.308** |
| FNR | Mediates transition from aerobic to anaerobic growth. | **1.226** |
| PurR | Regulates de novo synthesis of purine and pyrimidine nucleotides. | **1.205** |
| RcnR | Represses the expression of the cobalt and nickel metallo-regulator operon RcnAB. | **1.117** |
| Fur | Repressor of ferric uptake regulon. Controls expression of genes involved in iron homeostasis. | **0.962** |
| GlcC | Repressor of transport and catabolism of glycolate. | **-0.929** |
| Fis | DNA-bending protein that affects transcription of a wide variety of operons. | **-1.010** |
| Nac | Regulates genes involved in nitrogen metabolism under nitrogen-limiting conditions. | **-1.070** |
| GadW | Regulates expression of acid resistance genes *gadA* and *gadBC*. | **-1.202** |
| GadX | Activates genes involved in acid resistance, such as *gadA* and *gadBC*. | **-1.216** |
| FadR | Regulates fatty acid degradation while it activates fatty acid biosynthesis. | **-1.285** |
| LsrR | Represses transcription of the *lsr* operon, belongs to the quorum-sensing system. | **-1.489** |
| Sigma38 | RNA polymerase, sigma S factor. Genes associated with stationary phase. | **-1.690** |
| TorR | Activates the trimethilamine-N-oxide anaerobic respiratory system. | **-1.749** |
| MalT | Activator of the maltose regulon: uptake and catabolism of malto-oligosaccharides. | **-2.558** |

**Supplementary Table 3**. The top and bottom 10 *E. coli* K12 BW25113 regulators implicated in positive gene expression changes after 10 hours of growth in the presence of sublethal concentrations of tetrachloroauric acid, obtained using ISMARA. The z-score is a representation of differential expression induced in the target genes as a number of *n* standard deviations away from zero, with scores indicating up- (z-score > 0) or down- (z-score < 0) regulation in these genes.

| **Regulator** | **Description** | **Z-score** |
| --- | --- | --- |
| Fur | Repressor of ferric uptake regulon. Controls expression of genes involved in iron homeostasis. | **12.253** |
| MetJ | Represses the methionine operon. | **9.890** |
| PurR | Regulates de novo synthesis of purine and pyrimidine nucleotides. | **8.217** |
| TrpR | Expression of the tryptophan biosynthesis, regulation and transport regulon. | **7.936** |
| Zur | Zinc ABC transporter uptake system. | **6.075** |
| CueR | Controls the expression of the *copA* and *cueO* copper homeostasis genes. | **6.010** |
| ArgP | Modulates transcription of genes related to arginine and lysine metabolism. | **4.334** |
| Cra | Helps modulate the flow of central carbon metabolism. | **3.992** |
| ArgR | Represses the expression of arginine biosynthesis genes. | **3.748** |
| TyrR | Modulates the expression of genes involved in aromatic amino acid biosynthesis and transport. | **3.625** |
| TorR | Activates the trimethilamine-N-oxide anaerobic respiratory system. | **-5.169** |
| PrpR | Regulates the transcription of the propionate catabolism operon. | **-5.281** |
| Sigma32 | RNA polymerase, sigma H factor. Genes associated with response to heat shock. | **-5.475** |
| Sigma54 | RNA polymerase, sigma N factor. Genes associated with nitrogen-related processes. | **-6.327** |
| GlcC | Repressor of transport and catabolism of glycolate. | **-6.640** |
| FadR | Regulates fatty acid degradation while it activates fatty acid biosynthesis. | **-7.644** |
| CRP | Global activator of secondary carbon source pathways. | **-7.823** |
| MalT | Activator of the maltose regulon: uptake and catabolism of malto-oligosaccharides. | **-7.971** |
| LsrR | Represses transcription of the *lsr* operon, belongs to the quorum-sensing system. | **-8.440** |
| Sigma38 | RNA polymerase, sigma S factor. Genes associated with stationary phase. | **-11.918** |

**
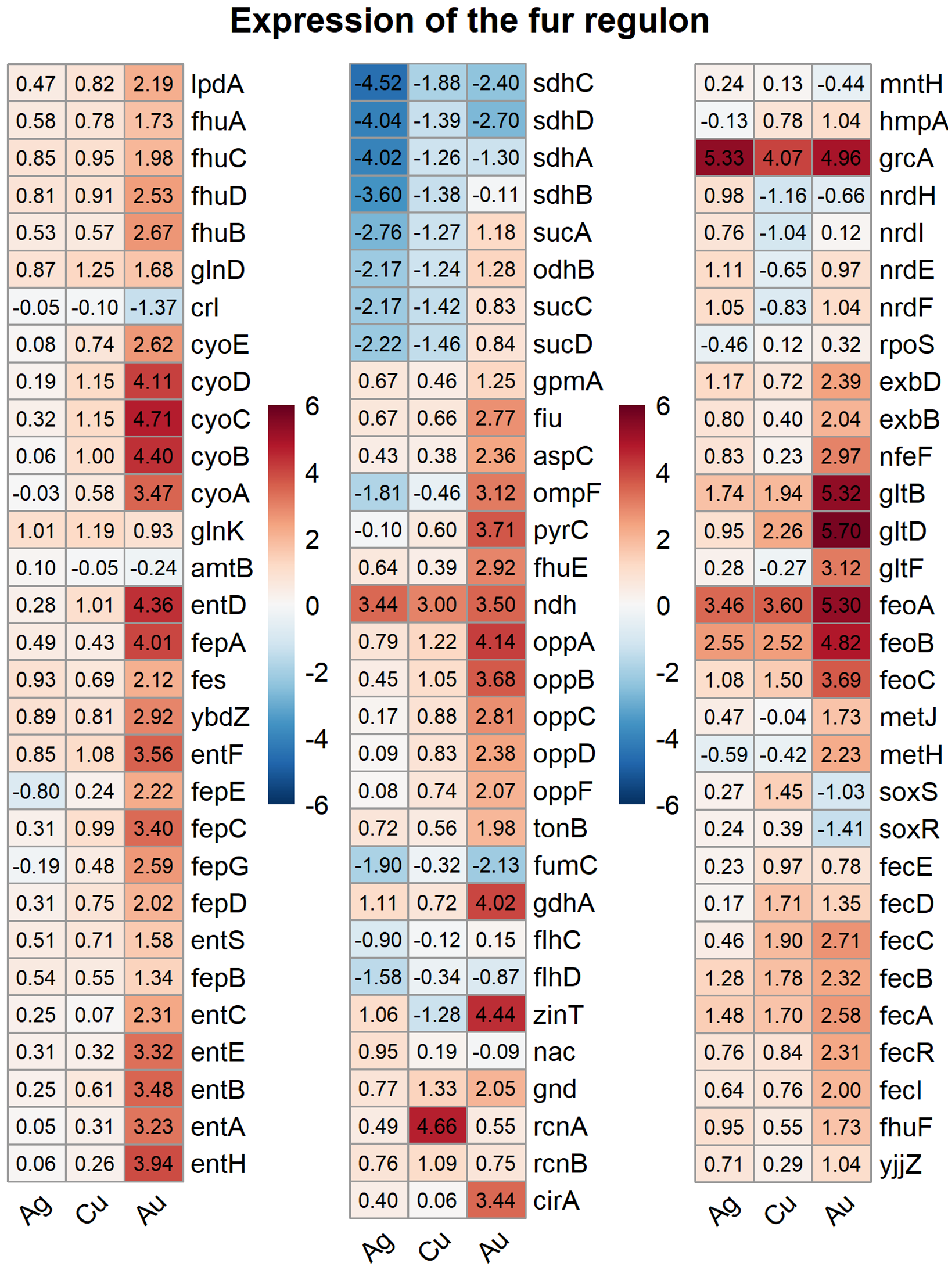
**

**Figure S11**. Average log_2_-fold change in gene expression for genes that are part of the Fur regulon. Each column corresponds to a different experimental condition: silver nitrate (Ag), copper sulfate (Cu), and tetrachloroauric acid (Au). Every experiment was contrasted against a non-metal challenge control, with three biological trials each.
